# First Morphological and Molecular Evidence of the Negative Impact of Diatom-Derived Hydroxyacids on the Sea Urchin *Paracentrotus lividus*

**DOI:** 10.1093/toxsci/kfw053

**Published:** 2016-03-16

**Authors:** Stefano Varrella, Giovanna Romano, Nadia Ruocco, Adrianna Ianora, Matt G. Bentley, Maria Costantini

**Affiliations:** *Department of Biology and Evolution of Marine Organisms; ^†^Department of Integrative Marine Ecology, Stazione Zoologica Anton Dohrn, Napoli, 80121, Italy;; ^‡^Faculty of Science and Technology, Bournemouth University, Dorset, BH12 5BB, United Kingdom

**Keywords:** diatoms, hydroxyacids, sea urchin, development, genes.

## Abstract

Oxylipins (including polyunsaturated aldehydes [PUAs], hydoxyacids, and epoxyalcohols) are the end-products of a lipoxygenase/hydroperoxide lyase metabolic pathway in diatoms. To date, very little information is available on oxylipins other than PUAs, even though they represent the most common oxylipins produced by diatoms. Here, we report, for the first time, on the effects of 2 hydroxyacids, 5- and 15-HEPE, which have never been tested before, using the sea urchin *Paracentrotus lividus* as a model organism. We show that HEPEs do induce developmental malformations but at concentrations higher when compared with PUAs. Interestingly, HEPEs also induced a marked developmental delay in sea urchin embryos, which has not hitherto been reported for PUAs. Recovery experiments revealed that embryos do not recover following treatment with HEPEs. Finally, we report the expression levels of 35 genes (involved in stress, development, differentiation, skeletogenesis, and detoxification processes) to identify the molecular targets affected by HEPEs. We show that the 2 HEPEs have very few common molecular targets, specifically affecting different classes of genes and at different times of development. In particular, 15-HEPE switched on fewer genes than 5-HEPE, upregulating mainly stress-related genes at a later pluteus stage of development. 5-HEPE was stronger than 15-HEPE, targeting 24 genes, mainly at the earliest stages of embryo development (at the blastula and swimming blastula stages). These findings highlight the differences between HEPEs and PUAs and also have important ecological implications because many diatom species do not produce PUAs, but rather these other chemicals are derived from the oxidation of fatty acids.

Several marine diatoms are rich in polyunsaturated fatty acids (PUFAs) and have traditionally been considered as an important food source for many aquatic animals. These fatty acids are also precursors for the production of toxic short-chain polyunsaturated aldehydes (PUAs) and other oxygenated fatty acid derivatives, collectively termed “oxylipins.” Production of all of these metabolites is triggered by cell damage or breakage, as during grazing or lysis of cells (Pohnert, 2005). Fatty acids liberated from cell membranes are oxidized by lipoxygenases (LOXs) to lipid hydroperoxides (FAHs), which are then rapidly converted within seconds to PUAs and other oxylipins. Of the known oxylipins, PUAs are the far best described and most comprehensively studied. This is due to PUAs being the first group described (Miralto *et al.*, 1999) and also are commercially available, inexpensive and sufficiently stable to allow for a range of laboratory bioassays to be conducted. PUAs have important biological and biochemical properties, disrupting a number of critical stages in reproductive and developmental processes including gametogenesis, gamete functionality, fertilization, embryonic mitosis, larval fitness, and competence in different marine invertebrates (Caldwell, 2009).

To date, very little information is available on other oxylipins because many of these compounds are extremely unstable, require direct isolation from the algal source material, and by default are neither readily available nor particularly amenable for biological testing. D’Ippolito *et al.* (2005) were the first to show that the diatom *Thalassiosira rotula* converted PUFAs into a variety of unprecedented oxylipins, as later confirmed by Fontana *et al.* (2007a). Fontana *et al.* (2007b) compared the effects of the well-known PUA-producing diatom *Skeletonema marinoi* (Miralto *et al.*, 1999; Ianora *et al.*, 2004) with 2 *Chaetoceros* (*C. similis* and *C. affinis*) species that did not produce PUAs, but which nonetheless impaired copepod hatching success. They showed that when *Chaetoceros* species were damaged, they produced fatty acid hydroperoxides (FAHs) and oxylipins such as hydroxyacids (HEPEs) and epoxyalcohols (HepETEs), as well as highly reactive oxygen species (ROS) of low acute toxicity to adult copepods but which depressed the viability of copepod gametes and offspring. These products are very similar with those produced as a wound-activated defense mechanism in terrestrial plants, suggesting that they are fundamental for the survival of plant cells and that they have been conserved through evolution. A major difference is in the precursor PUFAs, C16 and C20 fatty acids, used to synthesize these compounds in diatoms (d’Ippolito *et al.*, 2005; Pohnert, 2005) compared with the C18 fatty acids in terrestrial plants (Blée, 1998, 2002). Oxylipins formed in flowering plants include FAHs, hydroxyl- and keto-fatty acids, oxo-acids, epoxyalcohols, divinyl ethers, PUAs, and the plant hormones 12-oxo-phytodienoic acid and jasmonic acid (Andreou *et al.*, 2009), several of which have not yet been found in diatoms (e.g., jasmonic acid). Oxylipins are believed to play a pivotal role in plant defense because they act as chemical attractors (e.g., pheromones and pollinator attraction) or alarm signals against herbivore attack (e.g., in tritrophic interactions) and protective compounds (antibacterial and wound healing). Diatom oxylipins also show a high similarity to volatile organic carbons released from brown algae that are suggested to be involved in chemical signaling and pheromone attraction between gametes of different sexes (Andreou *et al.*, 2009). Several of these new compounds are also present in PUA producing species such as *T. rotula* (d’Ippolito *et al.*, 2005) and *S. marinoi* (Fontana *et al.*, 2007b), indicating that some diatoms produce both PUAs and these other oxylipins, whereas other species produce only these new metabolites. Two of these compounds, 15S-HEPE and threo-13,14-HepETE, have now also been reported by d’Ippolito *et al.* (2009) in the non–PUA-producing pennate diatom *Pseudo-nitzchia delicatissima*. The impact of a pool of HEPEs and HepETEs was tested on larval development in the copepod *Calanus helgolandicus* (Fontana *et al.*, 2007b). Nauplii showed incomplete development of swimming appendages with segments that differed from normal both in number and shape. Fluorescent images of the same specimen showed apoptotic regions corresponding to these morphological anomalies. These results indicated that PUAs were not the only class of molecules inducing malformations and apoptosis in copepods. Ianora *et al.* (2011) showed that 15S-HEPE was less biologically active when compared with the PUAs decadienal and heptadienal, but nonetheless reduced hatching success in copepods at high concentrations (20 µg ml ^−^ ^1 ^=^ ^100 μM) and induced apoptosis at 10 µg ml ^−^ ^1^ (corresponding to at about 50 μM). To our knowledge, these studies on copepods are the only ones performed so far to investigate the effect of diatom-derived oxylipins other than PUAs. Moreover, studies at the molecular level on the effects of these oxylipins on marine invertebrate development have never been conducted before.

Here, we test for the first time the effects of 2 common and abundant oxlipins produced by some marine diatoms, (±)-5-hydroxy-6E,8Z,11Z,14Z,17Z-eicosapentaenoic acid (henceforth 5-HEPE) and (±)-15-hydroxy-5Z,8Z,11Z,13E,17Z-eicosapentaenoic acid (henceforth 15-HEPE) (Cutignano *et al.*, 2011; d’Ippolito *et al.*, 2009; Fontana *et al.*, 2007a,b; Nanjappa *et al.*, 2014) on sea urchin *Paracentrotus lividus* development. Sea urchin embryos were treated with increasing concentrations of the 2 HEPEs to analyze morphological changes induced by exposure to these natural products and to define their mechanism of action and possible teratogenic activity. We also followed, using real-time quantitative PCR (qPCR), 35 genes belonging to different functional classes on *P. lividus* development to identify potential target genes of HEPEs.

## MATERIALS AND METHODS

*Ethics statement. Paracentrotus lividus* (Lamarck) sea urchins were collected from a location that is not privately owned or protected in any way, according to Italian legislation of the Marina Mercantile (Decreto del Presidente della Repubblica DPR 1639/68, 09/19/1980 confirmed on 01/10/2000). The field studies did not involve endangered or protected species. All animal procedures were in compliance with the guidelines of the European Union (Directive 609/86).

*Gamete collection, embryo culture, exposure to HEPEs*, *and morphological analysis*. Adult sea urchins of the species, *P. lividus*, were collected during the breeding season by scuba-diving in the Gulf of Naples, transported in an insulated box to the laboratory within 1 hour (h) after collection and maintained in tanks with circulating sea water until testing. Sea urchins were injected with 2 M KCl through the peribuccal membrane to obtain the emission of gametes. Eggs were washed with filtered sea water (FSW) and kept in FSW until use. Concentrated “dry” sperm was collected and kept undiluted at + 4 °C until use.

Eggs were fertilized in glass beakers in FSW, utilizing sperm-to-egg ratios of 100:1. We used this sperm-to-egg ratio after several tests to avoid polyspermy, so as to be certain that the effects on sea urchin embryos were due to the treatment with HEPEs. In fact, in the control embryos (embryos in FSW without HEPEs), we found a very low percentage of abnormal and delayed embryos (Figure 2), which represent the natural levels of developmental anomalies/delays in sea urchin embryos.

HEPEs were then added individually in the beakers containing the fertilized eggs in FSW 10 min after fertilization (minpf) at the following different concentrations: 6, 7, 8, 9, 10, 15, 30, 50, 70, and 90 µM. The HEPEs used in this work were as follows:

(±)-5-hydroxy-6E,8Z,11Z,14Z,17Z-eicosapentaenoic acid (Cayman Chemical, Ann Arbor, Michigan);

(±)-15-hydroxy-5Z,8Z,11Z,13E,17Z-eicosapentaenoic acid (Cayman Chemical).

Controls were also performed in FSW as described before, but without the addition of HEPEs.

Fertilized eggs were incubated at 20 °C in a controlled temperature chamber on a 12-h:12-h light:dark cycle. Experiments were conducted in triplicate using 3 egg groups collected from 3 different females. After 48 h of incubation, morphological malformations were determined for at least 200 plutei from each female (fixed in formaldehyde 4% in FSW) using a light microscope (Zeiss Axiovert 135TV; Carl Zeiss, Jena, Germany), in comparison with control embryos in FSW without HEPEs.

For recovery experiments, 3 concentrations were tested: 7, 10, and 15 µM. Embryos were treated with HEPEs as described above, then washed twice at different development times: 40 minpf and 2, 5, 9, and 24 h post fertilization (hpf). Embryos were grown to the pluteus stage. Controls were also performed, incubating embryos with HEPEs up to 48 hpf, without washing. The number of abnormal embryos was evaluated by fixing embryos in formaldehyde (4% in FSW) and counting under the light microscope.

To determine the stages that were most affected by HEPEs, eggs were fertilized and incubated without HEPEs, according to the procedure reported above. The development of embryos was followed by microscopic examination for different development times: 10 min before fertilization, 10 and 40 minpf, 2, 3, 5, and 9 hpf. HEPEs were then added at the same concentrations used for recovery experiments.

Statistical analysis was performed using GraphPad Prism version 4.00 for Windows (GraphPad Software, San Diego, California).

*RNA extraction and cDNA synthesis*. About 8000 eggs in 50 mL of FSW were fertilized and 7 μM of 5- and 15-HEPE were individually added at 10 mpf in different experiments. Samples were then collected at 5, 9, 24, and 48 hpf by centrifugation at 1800 relative centrifugal force for 10 min in a swing out rotor at 4 °C. The pellet was washed with phosphate buffered saline, and then frozen in liquid nitrogen and kept at −80 °C. Experiments were conducted in triplicate using 3 egg groups collected from 3 different females.

Total RNA was extracted from each developmental stage using TRIzol (Invitrogen; Life Technologies, Carlsbad, California) according to the manufacturer’s instructions. Contaminating DNA was degraded by treating each sample with a DNase RNase-free kit (Roche, Milan, Italy) according to the manufacturer’s instructions. The amount of total RNA extracted was estimated by the absorbance at 260 nm and the purity by 260/280 and 260/230 nm ratios, using a NanoDrop spectrophotometer (ND-1000 UV-Vis Spectrophotometer; NanoDrop Technologies, Wilmington, Delaware). The integrity of RNA was evaluated by agarose gel electrophoresis. Intact rRNA subunits (28S and 18S) were observed on the gel indicating minimal degradation of the RNA. For each sample, 600 ng of total RNA extracted was retrotranscribed with an iScript™ cDNA Synthesis kit (Bio-Rad, Milan, Italy), following the manufacturer’s instructions. Synthesized cDNA was used in real-time qPCR experiments without dilution.

To evaluate the efficiency of cDNA synthesis, a PCR was performed with primers of the reference gene, ubiquitin. The reaction was performed on the C1000 Touch Thermal Cycler (Applied Biosystem, Monza, Italy) in a final volume of 30 μl with 3 μl 10× PCR reaction buffer (Roche, Milan, Italy) in a final volume of, 3 μl 10× 2 mM dNTP, 1 μl 5 U/μl Taq (Roche, Milan, Italy), 100 ng/μl of each oligo, template cDNA and nuclease free water. The PCR program consisted of a denaturation step at 95 °C for 5 min, 35 cycles at 95 °C for 45 s, 60 °C for 1 min, and 72 °C for 30 s and a final extension step at 72 °C for 10 min.

*Gene expression by real-time qPCR*. For all real-time qPCR experiments, the data from each cDNA sample were normalized using ubiquitin mRNA as the endogenous control level according to Nemer *et al.* (1991; Romano *et al.*, 2011), the level of which remained relatively constant in all developmental stages examined. The expression level of 35 genes, previously analyzed in Varrella *et al.* (2014) (Supplementary Table 1) were followed by real-time qPCR.

Diluted cDNA was used as a template in a reaction containing a final concentration of 0.3 mM for each primer and 1× FastStart SYBR Green master mix (total volume of 10 μl) (Applied Biosystems, Monza, Italy). PCR amplifications were performed in a ViiATM7 Real Time PCR System (Applied Biosystems) thermal cycler using the following thermal profile: 95 °C for 10 min, 1 cycle for cDNA denaturation; 95 °C for 15 s and 60 °C for 1 min, 40 cycles for amplification; 72 °C for 5 min, 1 cycle for final elongation; 1 cycle for melting curve analysis (from 60 °C to 95 °C) to verify the presence of a single product. Each assay included a no-template control for each primer pair. To capture intra-assay variability, all real-time qPCR reactions were performed in triplicate. Fluorescence was measured using ViiATM7 software (Applied Biosystems). The expression of each gene was analyzed and internally normalized against ubiquitin using REST software (Relative Expression Software Tool, Weihenstephan, Germany) based on the Pfaffl method (Pfaffl, 2001; Pfaffl *et al.*, 2002). Relative expression ratios above 2 cycles were considered significant. Experiments were repeated at least twice. Experiments were conducted in triplicate using 3 egg groups collected from 3 different females. Statistical analysis was performed using GraphPad Prism version 4.00 for Windows (GraphPad Software).

## RESULTS

### Effects of HEPEs on Sea Urchin Development

Increasing concentrations (6, 7, 8, 9, 10, 15, 30, 50, 70, and 90 µM) of 5- and 15-HEPE were added separately on *P. lividus* embryos for 10 minpf. Neither of the 2 HEPEs arrested cell cleavage of sea urchin embryos up to a concentration of 90 µM. By contrast, the PUA decadienal blocked cell cleavage in a dose-dependent manner, with total arrest occurring at 5.26 µM (data from Romano *et al.*, 2010, Figure 1). Although HEPEs had no effect on cell cleavage, both induced malformations in developing embryos that principally affected the arms, spicules, and apex (shown in Supplementary Figure 1, in comparison with control embryos in FSW without HEPEs), similar to the malformations induced by PUAs (Marrone *et al.*, 2012; Varrella *et al.*, 2014). An increasing percentage of abnormal plutei was observed at HEPE concentrations ranging from 6 to 15 µM (Figure 2, top panel); decadienal also induced a dose-dependent effect on the generation of malformations but at much lower concentrations (data from Romano *et al.*, 2010). In addition to the presence of abnormal plutei, a small percentage of embryos showed delayed development at a concentration lower than 6 µM. The percentage of delayed embryos increased with increasing HEPE concentrations to become the only class of embryos present at the highest concentrations of 30 µM (Figure 2, top panel). Also in this case, the effect of HEPEs differed from the PUA decadienal for which no significant delay in development has been reported until now.

**FIG. 1.**
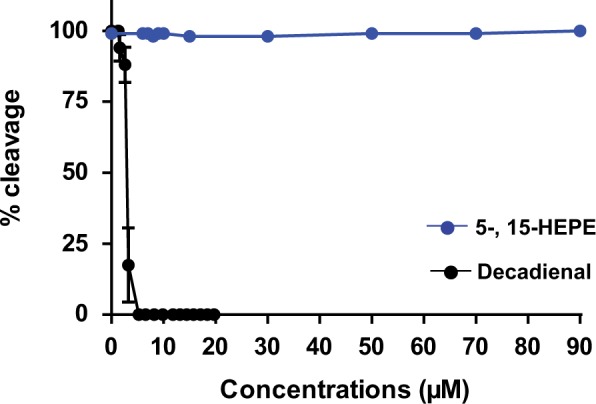
Cleavage inhibition in sea urchin embryos following decadienal (at the concentrations of 1.3, 1.6, 2.6, 3.3, 5.3, 6.6, 8.2, 9.9, 11.8, 13.1, 14.5, 15.8, 17.1, 18.4, and 19.7 μM; black line; data from Romano *et al.*, 2010) and 5- and 15-HEPE (at the concnetrations of 6, 7, 8, 9, 10, 15, 30, 50, 70 and 90 μM; blue line) treatments. Control is reported as 0 μM concentration. Abbreviation: HEPE, hydroxyacids.

**FIG. 2.**
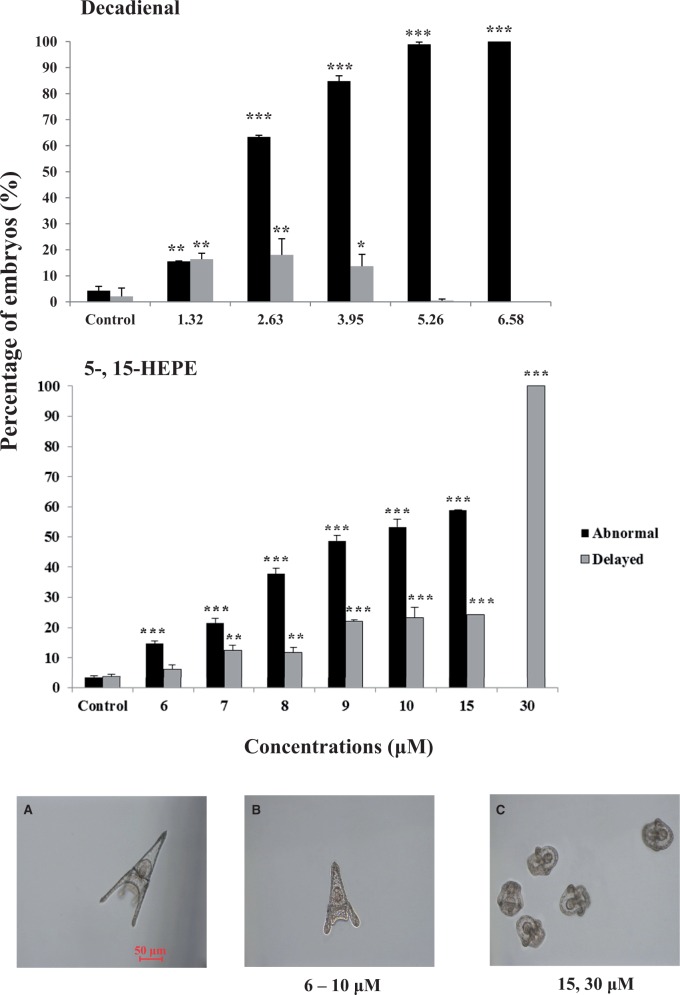
Abnormal and delayed embryos after HEPEs treatments. Top panel: percentage (%) of abnormal and delayed embryos when *Paracentrotus lividus* newly fertilized eggs were exposed to different concentrations of the polyunsaturated aldehydes decadienal (1.32, 2.63, 3.95, 5.26, and 6.58 μM; Romano *et al.*, 2010) and 5- and 15-HEPEs (6, 7, 8, 9, 10, 15, and 30 μM) at 48 h post fertilization (hpf). Significant differences (mean ± SD) compared with the control (4.3 ± 0.8 abnormal embryos; 3.3 ± 1.0 delayed embryos, data not shown): *P < .05, **P < .01, ***P < .001. One-way ANOVA (*P* < .05) with Tukey’s multiple comparison test. Experiments were conducted in triplicate using 3 egg groups collected from 3 different females. Bottom panel (A) control embryos (in filtered sea water) without HEPEs) at 48 hpf; (B) delayed embryos observed in samples treated with HEPEs at the concentrations from 6 to 10 µM and (C) delayed embryos observed in samples treated with HEPEs at 15 and 30 µM at 48 hpf. HEPE, hydroxyacids.

Furthermore, microscopic observations revealed that from 6 to 10 µM, the dose-dependent delay in the development of embryos was manifested by a shortening of the apex and arms: the morphology of the embryo closely resembling that of the control at the pluteus stage (Figure 2A, photo in bottom panel) and with only a slight reduction in body length (Figure 2B, photo bottom panel). At 15 and 30 µM, the development of embryos was much more delayed, with embryos still at the stage of early pluteus (Figure 2C, photo bottom panel).

The exposure time to HEPEs was extended to 1 week post fertilization (wpf) to follow the fate of plutei. After the pluteus stage, embryos began to retract their arms, assuming a pyramid shape (Supplementary Figure 2, embryo indicated with red arrow) and then a characteristic “ampoule-like” shape (Supplementary Figure 2, embryo indicated with white arrow). After 1 week of treatment with HEPEs (7, 10, 15, and 30 µM), the entire body was malformed (Supplementary Figs. 2B–H), and the arms were poorly retracted and degraded (Supplementary Figs. 2I–K). A significant number of delayed embryos were observed, still at the pluteus stage, and these were also malformed (Supplementary Figs. 2L and M). Supplementary Figure 3 reports the percentages of abnormal ampoules, abnormal plutei, normal ampoules, normal plutei, and early plutei in samples incubated with HEPEs at 7.0, 10.0, 15.0, and 30.0 µM detected at 1 wpf.

Recovery experiments were also performed to determine whether delayed and abnormal embryos were able to recover following treatment with HEPEs. The results indicated that delayed embryos were able to recover after exposure to 10 and 15 μM HEPE (Supplementary Figure 4A). On the contrary, the number of abnormal embryos remained almost the same as the unwashed embryos at 7.0, 10.0, and 15.0 μM (Supplementary Figure 4B), indicating that the malformations induced by HEPEs were not reversible.

To identify the developmental stage at which HEPEs affected embryonic development, HEPEs were added at different concentrations and at different development times. The results indicated that the most sensitive stages were between the 8-cell stage (2 hpf) and early blastula (5 hpf) (Supplementary Figure 5).

### Effects of HEPEs on Gene Expression

To better understand the morphological effects induced by HEPEs at the molecular level, *P. lividus* embryos were allowed to develop in the presence 7 µM concentrations of either 15-HEPE or 5-HEPE. Samples were collected at different development times after fertilization, corresponding to the stages of early blastula (5 hpf), swimming blastula (9 hpf), prism (24 hpf), and pluteus (48 hpf).

To identify potential gene targets of HEPEs, the expression levels of 35 genes were followed by real-time qPCR. These genes are stress related, involved in development and differentiation processes, and involved in detoxification and skeletogenic processes (Supplementary Figure 6). The control gene for real-time qPCR was ubiquitin (*UBIQ*); variation of expression levels was calculated as relative expression ratios of the analyzed genes with respect to control embryos in sea water without HEPEs. Only expression levels greater than 2-fold differences with respect to the controls were considered significant.

**FIG. 3.**
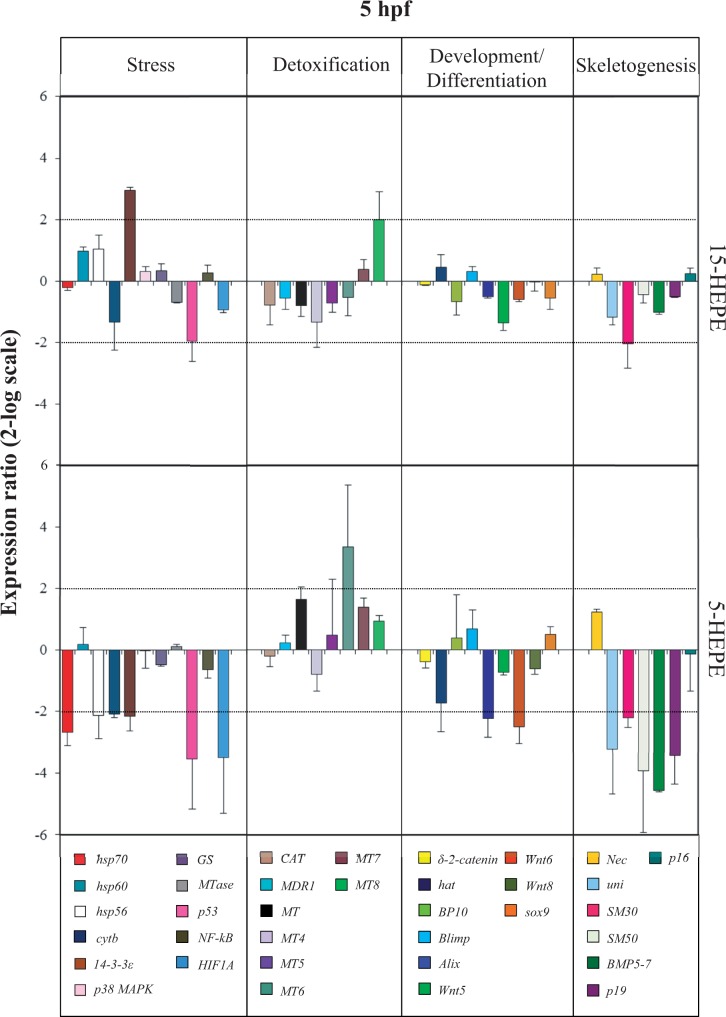
Real-time quantitative PCR (qPCR) at blastula stage. Histograms show the differences in expression levels of analyzed genes belonging to different functional classes (stress, detoxification, development and differentiation and skeletogenesis), followed by real time qPCR. *Paracentrotus lividus* embryos were grown in the presence of 15-HEPE and 5-HEPE at 7.0 µM and collected at 5 h post fertilization. Data are reported as a fold difference compared with control (mean ± SD) embryos in sea water without HEPEs. Fold differences greater than ±2 (see dotted horizontal guidelines at values of 2 and −2) were considered significant. Abbreviation: HEPE, hydroxyacids.

At the early blastula stage (5 hpf; Figure 3 and Supplementary Table 2), the 2 HEPEs differentially affected the expression levels of *14-3-3ε*. Whereas 15-HEPE upregulated this gene with 2.9-fold increase, 5-HEPE downregulated the same gene with a 2.2-fold decrease in expression levels. Another common target of 5- and 15-HEPE was the skeletogenic gene *SM30*, downregulated by both HEPEs by 2.1- and 2.2-fold, respectively, with respect to the control. Moreover, at this developmental stage, 5-HEPE downregulated the expression levels of another eleven genes: 5 stress genes, *hsp70, hsp56, cytb*, *p53*, and *HIF1A* (2.7-, 2.1-, 2.1-, 3.6-, and 3.5-fold, respectively), 2 genes involved in developmental and differentiation processes, *Wnt6* and *Alix* (2.5- and 2.2- fold, respectively), and another 4 skeletogenic genes, *uni, SM50, BMP5-7*, and *p19* (3.2-, 3.9-, 4.6-, and 3.4-fold, respectively). Only 1 metallothionein *MT6* was upregulated (3.3-fold increase).

**FIG. 4.**
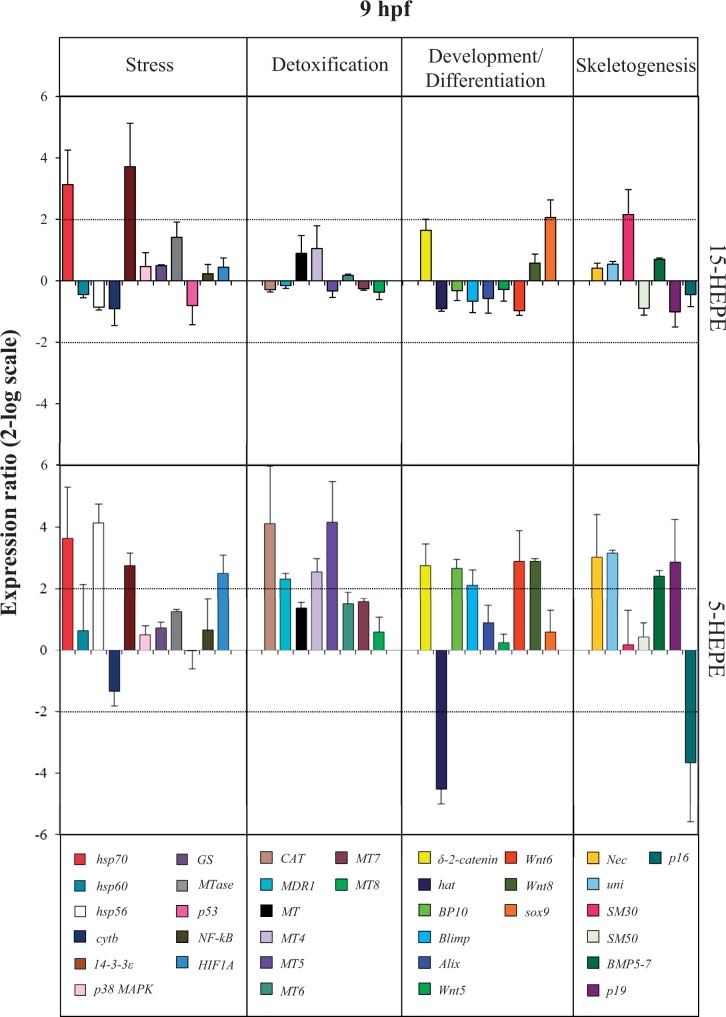
Real-time quantitative PCR (qPCR) a swimming blastula stage. Histograms show the differences in expression levels of analyzed genes, followed by real-time qPCR. *Paracentrotus lividus* embryos were grown in the presence of 7.0 µM of 15-HEPE and 5-HEPE and collected at 9 h post fertilization (for further details see also the legend to Figure 3). Abbreviation: HEPE, hydroxyacids.

At the swimming blastula stage (9 hpf; Figure 4 and Supplementary Table 2), 2 stress genes, *hsp70* and *14-3-3ε*, were common targets of the 2 HEPEs, both of which were upregulated: with 15-HEPE, there was a 3.1-fold increase for *hsp70* and 3.7-fold increase for *14-3-3ε*; with 5-HEPE ,there was a 3.6-fold increase for *hsp70* and 2.7-fold increase for *14-3-3ε*. At this developmental stage, 15-HEPE upregulated only 2 other genes, *sox9* and *SM30*, with a 2.1- and 2.2-fold increase with respect to the control. On the contrary, 5-HEPE affected the expression levels of another 17 genes. In particular, 2 stress genes, *hsp56* and *HIF1A*, respectively, showed a 4.1- and 2.5-fold increase in expression levels with respect to the control. Four genes involved in detoxification processes *CAT*, *MDR1*, *MT5*, and *MT4* were upregulated by 4.1-, 2.3-, 2.5-, and 4.1-fold, respectively. Six genes involved in the development and differentiation processes, *δ-2-catenin*, *BP10*, *Blimp*, *Wnt6*, and *Wnt8* were upregulated (2.8-, 2.6-, 2.1-, 2.9-, and 2.9-fold increase) and *hat* was downregulated (4.5-fold decrease); 5 skeletogenic genes (*Nec*, *uni*, *BMP5-7*, and *p19*) were upregulated by 3.0-, 3.1- 2.4-, and 2.9-fold and *p16* was downregulated by 3.7-fold.

**FIG. 5.**
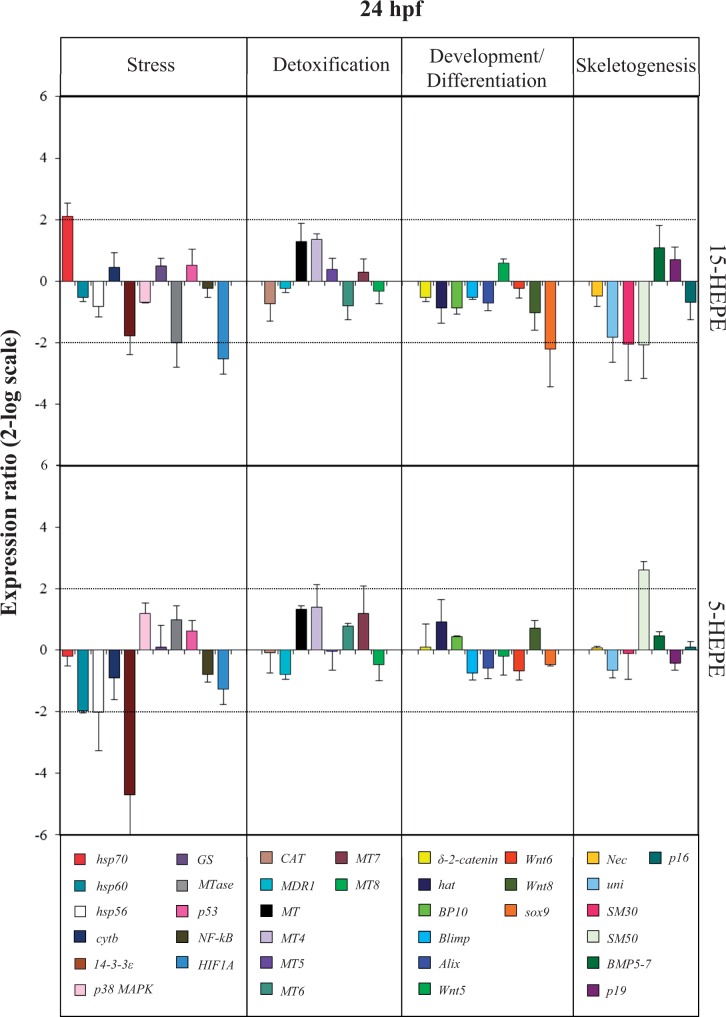
Real-time quantitative PCR (qPCR) at prism stage. Histograms show the differences in expression levels of analyzed genes, followed by real-time qPCR. *Paracentrotus lividus* embryos were grown in the presence of 7.0 µM of 15-HEPE and 5-HEPE and collected at 24 h post fertilization (for further details see also the legend to the Figure 3). Abbreviation: HEPE, hydroxyacids.

At the prism stage (24 hpf; Figure 5 and Supplementary Table 2), the 2 HEPEs differentially affected the expression level of the skeletogenic gene *SM50*. Whereas 15-HEPE downregulated this gene with a 2.1-fold decrease, 5-HEPE upregulated the same gene with a 2.6-increase in expression levels. 5-HEPE also had very few targets at this developmental stage: 2 stress genes *hsp70* (upregulated by 2.1-fold) and *HIF1A* (downregulated by 2.5-fold), and *sox-9* gene (downregulated by 2.2-fold). 15-HEPE also targeted another stress gene *14-3-3ε*, downregulating its expression level by 4.7-fold.

**FIG. 6.**
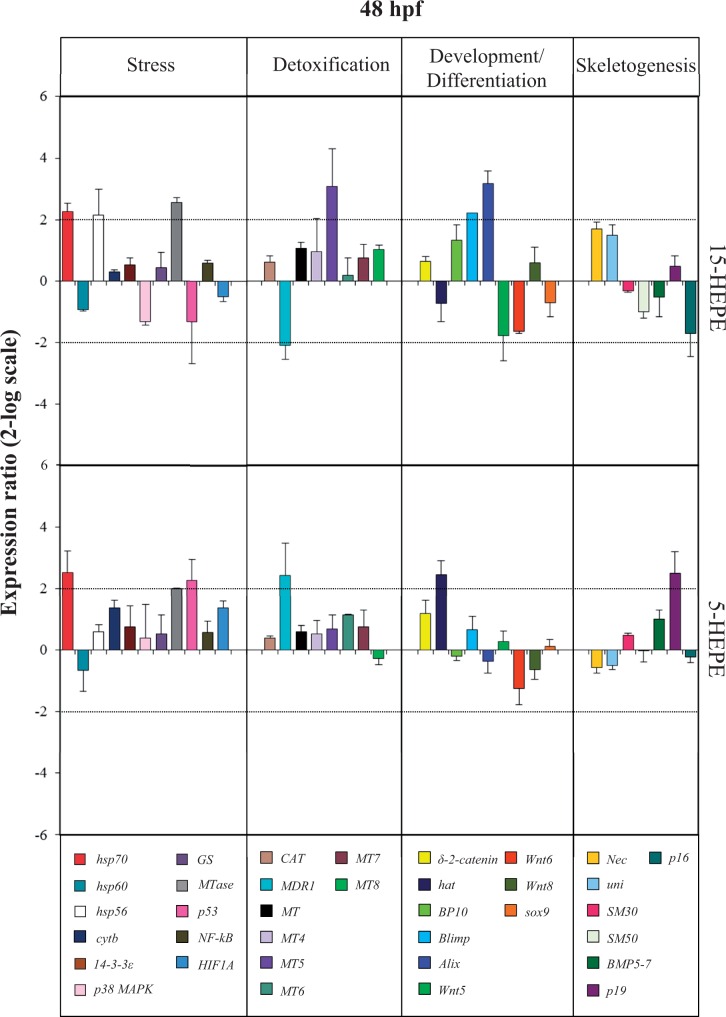
Real-time quantitative PCR (qPCR) at pluteus stage. Histograms show the differences in expression levels of analyzed genes, followed by real-time qPCR. *Paracentrotus lividus* embryos were grown in the presence of 7.0 µM of 15-HEPE and 5-HEPE and collected at 48 h post fertilization (for further details see also the legend to the Figure 3). Abbreviation: HEPE, hydroxyacids.

At the pluteus stage (48 hpf; Figure 6 and Supplementary Table 2), 2 genes were upregulated: the stress gene *hsp70* (2.3-fold for 15-HEPE and 2.5-fold for 5-HEPE) and *MDR1* (downregulated by 2.1-fold for 15-HEPE and upregulated by 2.4-fold for 5-HEPE). Moreover, 15-HEPE switched on 2 other stress genes, *hsp56* and *MTase* (2.2- and 2.6-fold, respectively); 2 genes involved in detoxification processes, *MDR1* and *MT5*, were also targeted (downregulated by 2.1-fold and upregulated by 3.1-fold); 2 genes involved in the development and detoxification processes, *Blimp* and *Alix* upregulated by 2.2- and 3.2-fold, respectively. 5-HEPE switched on *p53* with a 2.3-fold increase, the protease gene *hat* with 2.5-fold increase, and *p19* with 2.5-fold increase with respect to the control.

## DISCUSSION

Our results provide new morphological and molecular evidence of the toxic effects of diatom-derived hydroxyacids on the sea urchin *P. lividus*. Romano *et al.* (2013) had only shown that 15(S)-HEPE, 1 of the most abundant oxylipins produced by some diatoms (Cutignano *et al.*, 2006), inhibited the cleavage of *P. lividus* embryos at concentrations greater than 70 μM and induced 100% blockage of cellular divisions at 94 μM. Ianora *et al.* (2011) had shown that 15S-HEPE was less biologically active compared with the PUAs decadienal and heptadienal, but nonetheless reduced hatching success in copepods at concentrations greater than 100 μM and induced apoptosis at about 50 μM. Our new study confirms that hydroxyacids are able to induce malformations during sea urchin development and that these effects seem to be weaker than those induced by PUAs (Varrella *et al.*, 2014). The 2 HEPEs, 5- and 15-HEPE, induced similar morphological effects, with the production of the same percentage of abnormal plutei, depending on the concentration tested. An important finding of this study was that HEPEs were able to induce a marked delay in development. This effect has never been described before and was very evident at 30 μM concentrations (Figure 2), when all embryos at 48 hpf appeared morphologically similar and strongly delayed with respect to the control (embryos without HEPEs). These results suggest that a very specific morphological or molecular target is affected by HEPEs at this concentration. Of the PUAs tested, only octadienal delayed sea urchin development after 1 wpf (data not shown).

The results from the recovery experiments suggest that HEPEs exert a strong effect on embryonic development because the malformed embryos cannot recover after the induced damage, The most sensitive sea urchin stages to be affected by these compounds were between the 8-cell stage (2 hpf) and early blastula (5 hpf). This differs from PUAs that exert their effect mainly on the first stages of embryonic development. In fact, embryos do not recover after treatment with PUAs 10 mbf and/or 10 mpf, whereas later developmental stages do not seem to be affected (Varrella *et al.*, 2014).

**FIG. 7.**
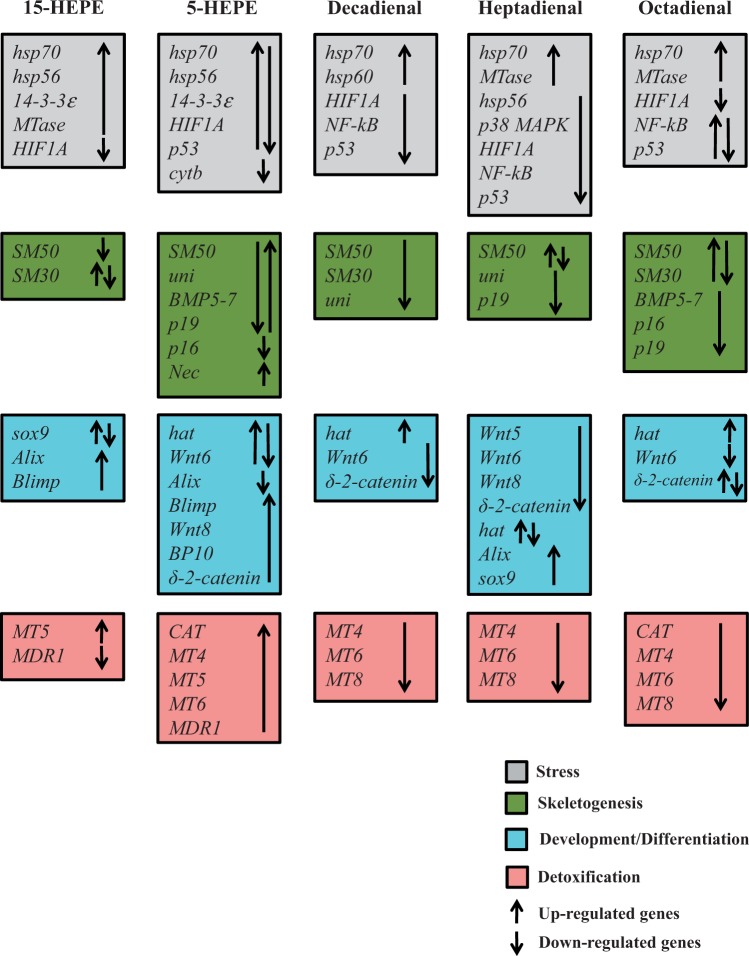
Synopsis of real time qPCR experiments. Synopsis of the patterns of up- and downregulation of different classes of genes in the sea urchin, *Paracentrotus lividus*, in the presence of HEPEs. PUAs decadienal, heptadienal, and octadienal are reported for the sake of comparison (Varrella *et al.*, 2014). Abbreviation: HEPE, hydroxyacids.

Our study also provides new information on the molecular effects of HEPEs on sea urchin embryos. We focused only on 2 HEPEs, 5- and 15-HEPE, because they are very common in the diatoms that have been studied until now (Cutignano *et al.*, 2006; d’Ippolito *et al.*, 2009; Fontana *et al.*, 2007a,b; Nanjappa *et al.*, 2014). The expression level of a great number of genes appeared to be modulated by these 2 HEPEs. A synopsis showing the patterns of up- and downregulation of different classes of genes is shown in Figure 7, in comparison with the same genes tested after sea urchins were treated with the 3 PUAs: decadienal, heptadienal, and octadienal.

Our results reveal that although the treatment with 5- and 15-HEPEs induced similar effects at the morphological level, the 2 HEPEs had very few common targets, specifically affecting different classes of genes and at different times of development. In particular, 15-HEPE switched on fewer genes than 5-HEPE, upregulating mainly stress-related genes at a later stage of development (at the pluteus stage, corresponding to 48 hpf). 5-HEPE was stronger than 15-HEPE because this hydroxyacid targeted 24 genes, mainly at the earliest stages of embryo development (at the blastula and swimming blastula stages, corresponding to 5 and 9 hpf). In both cases, HEPEs targeted all 4 functional classes (stress, development/differentiation, skeletogenic, and detoxification) of analyzed genes (Supplementary Figure 1).

Both HEPEs affected the expression levels of the canonical stress genes *hsp70* and *hsp56*, confirming that embryos were subjected to stress, activating these genes as a first defense system (Romano *et al.*, 2011). Several studies in higher invertebrates and vertebrates have reported the activation of protection systems by increasing the expression of heat shock proteins (Palotai *et al.*, 2008; Roccheri *et al.*, 2004) when exposed to stress, thereby enhancing cell survival under adverse environmental conditions (Diller, 2006). 5-HEPE also affected the expression level of the DNA methlytransferase gene *MTase*, which has been suggested to be a molecular marker in response to stress in invertebrates (Regev *et al.*, 1998). DNA methylation is an epigenetic mechanism that serves a wide variety of biological functions and plays an important role in gene regulation in response to environmental conditions (Garg *et al.*, 2015). In fact, the understanding of DNA methylation can provide insights in the regulatory mechanisms underlying stress response adaptation (Peng and Zhang, 2009). 5-HEPE also downregulated the expression levels of *cytb.* In the literature, there are no reports on the stress response of this gene.

Both HEPEs targeted expression levels of 14-3-3 epsilon. 14-3-3 proteins are a family of regulatory molecules able to bind functionally diverse signaling proteins, such as kinases and phosphatases (Russo *et al.*, 2010). Transcriptional increase and misexpression of 14-3-3 epsilon have been reported in sea urchin embryos exposed to UV-B (Russo *et al.*, 2010).

All 7 genes involved in skeletogenesis were targeted by both HEPEs. The expression levels of some of these skeletogenic genes have been shown to be affected also by manganese (Pinsino *et al.*, 2011) and by x-rays (Matranga *et al.*, 2010). In this context, a model has been proposed, linking univin, SM30, SM50, and nectin (Zito *et al.*, 2003). BMP5-7, belonging to a gene family reported as being positive regulators of oral and aboral ectoderm specifications (Duboc *et al.*, 2005; McIntyre *et al.*, 2013) was also affected by 5-HEPE. BMPs are required not only for skeletal patterning during embryonic development but also for bone response and remodeling to mechanical stimulation at specific anatomic sites in the skeleton (Ho *et al.*, 2008). 5-HEPE also affected the expression levels of *p16* and *p19*, 2 small acidic proteins involved in the formation of the biomineralized skeleton of sea urchin embryos and adults (Costa *et al.*, 2012). Recent studies have shown that these genes were also targeted by manganese and cadmium, confirming their important roles in skeletogenic processes (Migliaccio *et al.*, 2014).

Almost all genes involved in development and differentiation processes were affected by both HEPEs. *Alix* is a multifunctional protein involved in different cellular processes, including endocytic membrane trafficking, filamentous-actin remodeling, and cytokinesis (Romancino *et al.*, 2013). In sea urchins, this transcript encodes for a maternal protein involved in determination/differentiation events that is expressed from fertilization to the 2-cell stage. In sea urchin eggs, the gene is localized throughout the cytoplasm with a punctuated pattern, and soon after fertilization, it accumulates in the cytosol and in microvilli-like protrusions. *Blimp* gene encodes a protein that acts as a transcriptional repressor of beta-interferon gene expression. In sea urchin embryos, *Blimp* is itself linked into a feedback circuit that includes the wnt8 signaling ligand gene, and that this circuit generates an expanding torus of *blimp1* and *wnt8* expression (Smith and Davidson, 2008). *Hat*, affected by 5-HEPE at 9 hpf and, to a lower degree, at 5 hpf, is an early embryonic messenger transiently expressed during the blastula stage (Lepage *et al.*, 1992; Ghiglione et al., 1994). The *sox9* gene, targeted by 15-HEPE, is involved in left-right asymmetry processes (Duboc *et al.*, 2005). Of the genes belonging to the canonical Wnt pathway, *Wnt6* and *Wnt8* was targeted by 5-HEPE. This represents a very interesting result, supporting the essential role of Wnt6 in triggering endoderm specification (Croce *et al.*, 2011). *Wnt8* is associated with cell fate determination through canonical signaling pathways and is important for the morphogenetic movement of primary mesenchyme cells (Hammond and Hofmann, 2012; Smith and Davidson, 2008). Decrease of expression levels of this gene was also detected in *S. purpuratus* embryos exposed to low CO_2_ levels (Todgham and Hofmann, 2009).

BP10 is a metalloprotease of the astacin family, secreted at the blastula stage by sea urchin embryos (Lhomond *et al.*, 2012). The transcription of this gene (targeted by 5-HEPE) is transiently activated around the 16- to 32-cell stage, and the accumulation of its transcript is limited to a short period at the blastula stage (Lepage *et al.*, 1992).

Five genes involved in detoxification processes were targeted by both HEPEs. The gene *MDR1* belongs to ATP-binding cassette transporters, which are activated by sub-lethal doses of specific contaminants (such as oxybenzone, mercuric chloride, and tributyltin) during embryonic development (from the zygote to the blastula stage) of sea urchins (Bošnjak *et al.*, 2013). Moreover, sea urchin embryos utilize this gene in cell signaling and lysosomal and mainly mitochondrial homeostasis (Shipp and Hamdoun, 2012). Ragusa *et al.* (2013) reported that 2 metallothionein genes (*MT7* and *MT8*) appeared to be constitutively expressed and upregulated upon cadmium treatment, whereas other genes (*MT4*, *MT5*, and *MT6*) were not transcribed in control embryos and were specifically activated in response to cadmium treatment. A downregulation of *MT6* was also found in *P. lividus* embryos after cadmium treatments, whereas *MT4* was upregulated in response to manganese (Migliaccio *et al.*, 2014). *CAT* gene is a key antioxidant enzyme in the defense against oxidative stress, and its activation by 5-HEPE suggests the activation of a specific detoxification system in sea urchins only after exposure to this hydroxyacid. Under the same experimental conditions used in the present work, caspase 3/7 and caspase-8 were also molecular targets of 5-HEPEs (Ruocco *et al.* under revision). These genes play a central role in cell apoptosis processes indicating that HEPEs may activate the apoptotic machinery of cells.

Taken together, these molecular results are in accordance with our morphological results indicating that the majority of malformations affected the skeleton and the plan of development and differentiation of sea urchin embryos, as reported in Figure 7. In fact, several genes belonging to the skeletogenic, developmental, and differentiation classes were affected by HEPEs.

Very recently, we demonstrated that all *P. lividus* genes analyzed in this work, as possible targets of HEPEs, were intercorrelated (Varrella et al., 2016). We performed interactomic analysis by Ingenuity Pathway Analysis Version 7.1 (IPA, Ingenuity Systems, Inc, Redwood City, California) to identify relationships between the 35 *P. lividus* genes analyzed here by real-time qPCR (Figs. 3–6) on the basis of associated functions and data mining from experimental studies reported in the literature. Our data indicated that these 35 genes were functional correlated with 4 HUB genes (including *NF-κB*, *p53*, *δ-2-catenin*, and *HIF1A*), viewed as important nodes in the ne2rk with the largest degrees (i.e., nodes that share the largest number of connections with the other nodes). We also proposed a working model of hypothetical pathways potentially involved in the stress response to toxic oxylipins (Varrella *et al.*, 2016). According to this model, oxylipins initially downregulated (probably through transmembrane receptors) the 4 nuclear HUB genes: they initially downregulated δ-2-*catenin*, which regulates *NF-κB*, and then *HIF1A*, which in turn is regulated by *p53*; *p53* and *NF-κB* regulate each other. On the other hand, oxylipins may also affect some of these 35 genes, having a key role in different functional responses, such as stress, development, differentiation, and detoxification. A cross talk is possible between HUB genes and the targeted genes. All these genes may drive sea urchin embryos toward teratogenesis and/or apoptosis (or cell death), depending on the oxylipins exposure dose. Furthermore, in our previous study, we demonstrate that *P. lividus* places in motion these different classes of genes, to defend itself against the PUAs decadienal, heptadienal, and octadienal (Marrone *et al.*, 2012; Varrella *et al.*, 2014). In fact, the need to deal with physical, chemical, and biological challenges has driven the evolution of an array of gene families and pathways affording protection from, and repair of, damage to stress. Genes and proteins affording such protection for an organism collectively may be considered a “defensome,” as reported for the sea urchin *S. purpuratus* (Goldstone *et al.*, 2006). The defensome is an integrated ne2rk of genes and pathways, which allow an organism to mount an orchestrated defense against toxicants, including microbial products, heavy metals, phytotoxins, and other natural compounds. We can therefore hypothesize that the genes responsive to PUAs and HEPEs can be considered as a part of the chemical defensome or stress surveillance system of the sea urchin *P. lividus*, affording protection from environmental toxicants

To date, most studies have reported on the effects of diatom extracts or purified toxins on marine organisms, but data from in vivo exposure to intact diatoms are scarce. Very recently, Gudimova *et al.* (2016) have conducted sea urchin egg incubation and plutei feeding experiments to test whether intact diatom cells affected sea urchin embryo development and survival. The common northern sea urchins *Strongylocentrotus droebachiensis* and *Echinus acutus* were exposed to northern strains of the diatoms *C. socialis*, *S. marinoi*, *C. furcellatus*, *Attheya longicornis*, *Thalassiosira* *gravida*, and *Porosira glacialis*. Sea urchin egg hatching and embryogenesis were inhibited by the intact diatom cell suspensions. *Skeletonema* *marinoi* resulted the most potent species, causing acute mortality in *S. droebachiensis* eggs after only 4-h exposure to high (50 mg/L Chla) diatom concentrations, as well as 24-h exposure to normal (20 mg/L Chla) and high diatom concentrations. The second most potent species was *T. gravida* that caused acute mortality after 24-h exposure to both diatom concentrations. *Attheya* *longicornis* was the least harmful of the diatom species in terms of embryo development arrest, and it was the species that was most actively ingested by *S. droebachiensis* plutei, suggesting that some sea urchins may be able to detect the presence of oxylipins and avoid feeding on toxic cells.

In conclusion, our study provides a first insight to understanding the morphological and molecular effects of diatom-derived hydroxyacids on sea urchins. To date, most of the negative effects of diatoms on invertebrate development has been attributed to the production of PUAs (reviewed by Caldwell, 2009; Ianora and Miralto, 2010; Romano *et al.*, 2010, 2011; Varrella *et al.*, 2014;). Our results indicate, for the first time, that HEPEs can also affect the developmental program in sea urchin embryos and in different ways compared with PUAs. In fact, PUAs are able to induce malformations and teratogenesis at low concentrations and apoptosis at higher concentrations (Romano *et al.*, 2010) compared with HEPEs, which induce the same effects but at higher concentrations. By contrast, HEPEs are also able to induce delay during embryo development which has not been reported before for PUAs. Future efforts in toxicological studies should therefore be directed to better understanding the cellular mechanisms underlying the responses of marine benthic organisms to toxic diatom-derived oxylipin exposure.

Given the importance of diatom blooms in nutrient-rich aquatic environments, our findings have interesting ecological implications, considering that hydroxyacids represent one of the most common classes of oxylipins produced by some diatoms, much more common than the better-known PUAs. In aquatic ecosystems, a considerable proportion of the primary production from phytoplankton bloom sinks to the sediment (Lignell *et al.*, 1993; Wetzel 1995). Most benthic communities below the photic zone are entirely dependent on such imported organic matter (Graf 1992). Sea urchins and their larvae may come in contact with diatom PUAs in the field at the end of a bloom, with the mass sinking of diatoms to the sediment (Vanaverbeke *et al.*, 2008). Because they are browsing animals that eat phytoplankton and organic matter in the sand or mud, sea urchins may accumulate oxylipins through feeding or be exposed to high local concentrations of these compounds that may affect growth performance, as already demonstrated for copepods exposed to PUAs (reviewed by Ianora and Miralto, 2010). Not much is known on ingestion rates of sea urchins. Fenaux *et al.* (1985) calculated ingestion rates of pluteus larvae of the sea urchin *Paracentrotus lividus* (corresponding to our pluteus stages after 48 h) fed on a diet of 2 algae, *Hymenomonas elongata* (cell diameter 8.0–13.5 µm) and *Monocrysis lutheri* (cell diameter 3.7–4.2 µm) that were 0.67 × 10^5^ µm^3^ pluteus ^−^ ^1^ days ^−^ ^1^ and 1.88 × 10^5^ µm^3^ pluteus ^−^ ^1^ days-1, respectively. To simulate ingestion rates of HEPEs necessary to induce variations in gene expression levels in our study, we used as a model the oxylipin-producing diatom species *Skeletonema marinoi*. Because the size of *S. marinoi* is intermediate between the other 2 cell types (6–8 µm), we assumed ingestion rates on this diatom of about 1.0 × 10^5^ µm^3^ pluteus ^−^ ^1^ days ^−^ ^1^. Considering that Cutignano *et al.* (2011) reported concentrations of 0.22 pg cell ^−^ ^1^ of HEPEs during the spring bloom in the Adriatic Sea in 2005, we calculated daily ingestion rates of HEPEs as (1.0 × 10^5^ µm^3^ pluteus ^−^ ^1^ days ^−^ ^1^) × 0.22 pg cell ^−^ ^1 ^=^ ^22 000 pg (= 0.022 µg). Hence, sea urchins would need to ingest this concentration for 100 days (about 3 months) in order to accumulate 2.2 μg ml ^−^ ^1^ (corresponding to 7 μM) HEPEs used in our experiments. This is not unfeasible because diatom blooms dominated by *S. marinoi* can last from February to May in the North Adriatic Sea (Ianora *et al.*, 2004). Therefore, we conclude that the concentrations here tested are well within the significant range for affecting growth and performance of sea urchins during bloom conditions.

## SUPPLEMENTARY DATA

Supplementary data are available online at http://toxsci.oxfordjournals.org/.

Supplementary Data

## References

[kfw053-B1] Andreou A. Brodhun F. Feussner I. (2009). Biosynthesis of oxylipins in non-mammals. Prog. Lipid Res. 48, 148–170. 19268690 10.1016/j.plipres.2009.02.002

[kfw053-B2] Blée E. (1998). Phytooxylipins and plant defense reactions. Prog. Lipid Res. 37, 33–72. 9764311 10.1016/s0163-7827(98)00004-6

[kfw053-B3] Blée E. (2002). Impact of phyto-oxylipins in plant defense. Trends Plant Sci. 7, 315–322. 12119169 10.1016/s1360-1385(02)02290-2

[kfw053-B4] Bošnjak I. Zaja R. Klobučar R. S. Šver L. Franekić J. Smital T. (2013). Identification of ABC transporter genes in gonad tissue of two mediterranean sea urchin species: Black, *Arbacia lixula* L., and rocky, *Paracentrotus lividus* L. Bull. Envir. Contam. Toxicol. 91, 415–419. 10.1007/s00128-013-1021-8 23744482

[kfw053-B5] Caldwell G. S. (2009). The influence of bioactive oxylipins from marine diatoms on invertebrate reproduction and development. Mar. Drugs 7, 367–400. 19841721 10.3390/md7030367 PMC2763107

[kfw053-B6] Costa C. Karakostis K. Zito F. Matranga V. (2012). Phylogenetic analysis and expression patterns of p16 and p19 in *Paracentrotus lividus* embryos. Dev. Genes Evol. 222, 245–251. 22565340 10.1007/s00427-012-0405-9

[kfw053-B7] Croce J. Range R. Wu S. Y. Miranda E. Lhomond G. Peng J. C. Lepage T. McClay D. R. (2011). Wnt6 activates endoderm in the sea urchin gene regulatory network. Development 138, 3297–3306. 21750039 10.1242/dev.058792 PMC3133919

[kfw053-B8] Cutignano A. d’Ippolito G. Romano G. Lamari N. Cimino G. Febbraio F. Nucci R. Fontana A. (2006). Chloroplastic glycolipids fuel aldehyde biosynthesis in the marine diatom *Thalassiosira rotula.* Chembiochem 7, 450–456. 16470764 10.1002/cbic.200500343

[kfw053-B9] Cutignano A. Lamari N. d’Ippolito G. Manzo E. Cimino G. Fontana A. (2011). Lypoxygenase products in marine diatoms: a concise analytical method to explore the functional potential of oxylipins. J. Phycol. 47, 233–243. 27021855 10.1111/j.1529-8817.2011.00972.x

[kfw053-B10] Diller K. R. (2006). Stress protein expression kinetics. Ann. Rev. Biomed. Eng. 8, 403–424. 16834562 10.1146/annurev.bioeng.7.060804.100449

[kfw053-B11] d’Ippolito G. Cutignano A. Briante R. Febbraio F. Cimino G. Fontana A. (2005). New C 16 fatty-acid-based oxylipin pathway in the marine diatom *Thalassiosira rotula.* Organic Biomol. Chem. 3, 4065–4070. 10.1039/b510640k 16267584

[kfw053-B12] d’Ippolito G. Lamari N. Montresor M. Romano G. Cutignano A. Gerecht A. Cimino G. Fontana A. (2009). 15S-lipoxygenase metabolism in the marine diatom *Pseudo-nitzschia delicatissima.* New Phytol. 183, 1064–1071. 19538551 10.1111/j.1469-8137.2009.02887.x

[kfw053-B13] Duboc V. Röttinger E. Lapraz F. Besnardeau L. Lepage T. (2005). Left-right asymmetry in the sea urchin embryo is regulated by nodal signaling on the right side. Dev. Cell 9, 147–158. 15992548 10.1016/j.devcel.2005.05.008

[kfw053-B14] Fenaux L. Cellario C. Etienne M. (1985). Variations in the ingestion rate of algal cells with morphoòogical development of larvae of Paracentrotus lividus (Echinodermata: Echinoidea). Mar. Ecol. Prog. Ser. 24, 161–165.

[kfw053-B15] Fontana A. d’Ippolito G. Cutignano A. Miralto A. Ianora A. Romano G. Cimino G. (2007a). Chemistry of oxylipin pathways in marine diatoms. Pure Appl. Chem. 79, 481–490.

[kfw053-B16] Fontana A. d’Ippolito G. Cutignano A. Romano G. Lamari N. Massa Gallucci A. Cimino G. Miralto A. Ianora A. (2007b). A. LOX-induced lipid peroxidation mechanism responsible for the detrimental effect of marine diatoms on zooplankton grazers. Chembiochem 8, 1810–1818. 17886321 10.1002/cbic.200700269

[kfw053-B17] Garg R. Chevala N. V. V. S. Shankar R. Jain M. (2015). Divergent DNA methylation patterns associated with gene expression in rice cultivars with contrasting drought and salinity stress response. Sci. Rep. 5, 14922. 26449881 10.1038/srep14922 PMC4598828

[kfw053-B18] Ghiglione C. Lhomond G. Lepage T. Gache C. (1994). Structure of the sea urchin hatching enzyme gene. Eur. J. Biochem. 219, 845–854. 8112336 10.1111/j.1432-1033.1994.tb18566.x

[kfw053-B19] Goldstone J. V. Hamdoun A. Colle B. J. Howard-Asby M. Nebert D. W. Scally M. Dean M. Epel D. Hahn M. E. Stegeman J. J. (2006). The chemical defensome: environmental sensing and response genes in the *Strongylocentrotus purpuratus* genome. Dev. Biol. 300, 366–384. 17097629 10.1016/j.ydbio.2006.08.066 PMC3166225

[kfw053-B20] Graf G. (1992). Benthic-pelagic coupling: a benthic view. Oceanography and Marine Biology. Ann. Rev. 30, 149–190.

[kfw053-B21] Gudimova E. Eilertsen H. C. Jorgensen T. O. Hansen E. (2016). In vivo exposure to northern diatoms arrests sea urchin embryonic development. Toxicon 109, 63–69. 26559615 10.1016/j.toxicon.2015.11.001

[kfw053-B22] Hammond L. M. Hofmann G. E. (2012). Early developmental gene regulation in *Strongylocentrotus purpuratus* embryos in response to elevated CO2 seawater conditions. J. Exp. Biol. 215, 2445–2454. 22723484 10.1242/jeb.058008

[kfw053-B23] Ho A. M. Marker P. C. Peng H. Quintero A. J. Kingsley D. M. Huard J. (2008). Dominant negative Bmp5 mutation reveals key role of BMPs in skeletal response to mechanical stimulation. BMC Dev. Biol. 8, 35. 18380899 10.1186/1471-213X-8-35 PMC2335095

[kfw053-B24] Ianora A. Miralto A. (2010). Toxigenic effects of diatoms on grazers, phytoplankton and other microbes: a review. Ecotoxicology 19, 493–511. 19924531 10.1007/s10646-009-0434-y

[kfw053-B25] Ianora A. Miralto A. Poulet S. A. (2004). Aldehyde suppression of copepod recruitment in blooms of a ubiquitous planktonic diatom. Nature 429, 403–407. 15164060 10.1038/nature02526

[kfw053-B26] Ianora A. Romano G. Carotenuto Y. Esposito F. Roncalli V. Buttino I. Miralto A. (2011). Impact of the diatom oxylipin 15S-HEPE on the reproductive success of the copepod *Temora stylifera.* Hydrobiologia 666, 265–275.

[kfw053-B27] Lepage T. Sardet C. Gache C. (1992). Spatial expression of the hatching enzyme gene in the sea urchin embryo. Dev. Biol. 150, 23–32. 1537434 10.1016/0012-1606(92)90004-z

[kfw053-B28] Lhomond G. McClay D. R. Gache C. Croce J. C. (2012). Frizzled 1/2/7 signaling directs β-catenin nuclearisation and initiates endoderm specification in macromeres during sea urchin embryogenesis. Development 139, 816–825. 22274701 10.1242/dev.072215 PMC3265065

[kfw053-B29] Lignell R. Heiskanen A. S. Kuosa H. Gundersen K. Kuuppo-Leinikki P. Pajuniemi R. Uitto A. (1993). Fate of a phytoplankton spring bloom: sedimentation and carbon flow in the planktonic food web in the northern Baltic. Mar. Ecol. Prog. Ser. 94, 239–252.

[kfw053-B30] Marrone V. Piscopo M. Romano G. Ianora A. Palumbo A. Costantini M. (2012). Defensome against toxic diatom aldehydes in the sea urchin *Paracentrotus lividus.* PLoS One 7, e31750. 22363721 10.1371/journal.pone.0031750 PMC3282763

[kfw053-B31] Matranga V. Zito F. Costa C. Bonaventura R. Giarrusso S. Celi F. (2010). Embryonic development and skeletogenic gene expression affected by X-rays in the Mediterranean sea urchin *Paracentrotus lividus.* Ecotoxicology 19, 530–537. 19943107 10.1007/s10646-009-0444-9

[kfw053-B32] McIntyre D. C. Seay N. W. Croce J. C. McClay D. R. (2013). Short-range Wnt5 signaling initiates specification of sea urchin posterior ectoderm. Development 140, 4881–4889. 24227654 10.1242/dev.095844 PMC3848187

[kfw053-B33] Migliaccio O. Castellano I. Romano G. Palumbo A. (2014). Stress response to cadmium and manganese in *Paracentrotus lividus* developing embryos is mediated by nitric oxide. Aquat. Toxicol. 156, 125–134. 25181703 10.1016/j.aquatox.2014.08.007

[kfw053-B34] Miralto A. Barone G. Romano G. Poulet S. A. Ianora A. Russo G. L. Buttino I. Mazzarella G. Laabir M. Cabrini M., and., (1999). The insidious effect of diatoms on copepod reproduction. Nature 402, 173–176.

[kfw053-B35] Nanjappa D. D’Ippolito G. Gallo C. Zingone A. Fontana A. (2014). Oxylipin diversity in the diatom family leptocylindraceae reveals DHA derivatives in marine diatoms. Mar. Drugs 12, 368–384. 24445306 10.3390/md12010368 PMC3917278

[kfw053-B36] Nemer M. Rondinelli E. Infante D. Infante A. A. (1991). Polyubiquitin RNA characteristics and conditional induction in sea urchin embryos. Dev. Biol. 145, 255–265. 1645680 10.1016/0012-1606(91)90124-l

[kfw053-B37] Palotai R. Szalay M. S. Csermely P. (2008). Chaperones as integrators of cellular networks: changes of cellular integrity in stress and diseases. IUBMB Life 60, 10–18. 18379988 10.1002/iub.8

[kfw053-B38] Peng H. Zhang J. (2009). Plant genomic DNA methylation in response to stresses: Potential applications and challenges in plant breeding. Progr. Natural Sci. 19, 1037–1045.

[kfw053-B39] Pfaffl M. W. (2001). A new mathematical model for relative quantification in real-time RT-PCR. Nucleic Acids Res. 29, e45. 11328886 10.1093/nar/29.9.e45 PMC55695

[kfw053-B40] Pfaffl M. W. Horgan G. W. Dempfle L. (2002). Relative expression software tool (REST) for group-wise comparison and statistical analysis of relative expression results in real-time PCR. Nucleic Acids Res. 30, e36. 11972351 10.1093/nar/30.9.e36 PMC113859

[kfw053-B41] Pinsino A. Roccheri M. C. Costa C. Matranga V. (2011). Manganese interferes with calcium, perturbs ERK signaling, and produces embryos with no skeleton. Toxicol. Sci. 23, 217–230. 21659617 10.1093/toxsci/kfr152

[kfw053-B42] Pohnert G. (2005). Diatom/copepod interactions in plankton: the indirect chemical defense of unicellular algae. Chembiochem 6, 946–959. 15883976 10.1002/cbic.200400348

[kfw053-B43] Ragusa M. A. Costa S. Gianguzza M. Roccheri M. C. Gianguzza F. (2013). Effects of cadmium exposure on sea urchin development assessed by SSH and RT-qPCR: Metallothionein genes and their differential induction. Mol. Biol. Rep. 40, 2157–2167. 23212613 10.1007/s11033-012-2275-7

[kfw053-B44] Regev A. Lamb M. J. Jablonka E. (1998). The role of DNA methylation in invertebrates: developmental regulation or genome defense. Mol. Biol. Evol. 15, 880–891.

[kfw053-B45] Roccheri M. C. Agnello M. Bonaventura R. Matranga V. (2004). Cadmium induces the expression of specific stress proteins in sea urchin embryos. Biochem. Biophys. Res. Commun. 321, 80–87. 15358218 10.1016/j.bbrc.2004.06.108

[kfw053-B46] Romancino D. P. Anello L. Morici G. d’Azzo A. Bongiovanni A. Di Bernardo M. (2013). Identification and characterization of PlAlix, the Alix homologue from the Mediterranean sea urchin *Paracentrotus lividus.* Dev. Growth Diff. 55, 237–246. 10.1111/dgd.12023 23302023

[kfw053-B47] Romano G. Costantini M. Buttino I. Ianora A. Palumbo A. (2011). Nitric oxide mediates the stress response induced by diatom aldehydes in the sea urchin *Paracentrotus lividus.* PLoS One 6, e25980. 22022485 10.1371/journal.pone.0025980 PMC3191173

[kfw053-B48] Romano G. Manzo E. Russo G. L. d’Ippolito G. Cutignano A. Russo M. Fontana A. (2013). Design and synthesis of pro-apoptotic compounds inspired by diatom oxylipins. Mar. Drugs 11, 4527–4543. 24232667 10.3390/md11114527 PMC3853743

[kfw053-B49] Romano G. Miralto A. Ianora A. (2010). Teratogenic effects of diatom metabolites on sea urchin *Paracentrotus lividus* embryos. Mar. Drugs 8, 950–967. 20479962 10.3390/md8040950 PMC2866470

[kfw053-B50] Russo R. Zito F. Costa C. Bonaventura R. Matranga V. (2010). Transcriptional increase and misexpression of 14-3-3 epsilon in sea urchin embryos exposed to UV-B. Cell Stress Chaperones 15, 993–1001. 20607471 10.1007/s12192-010-0210-1 PMC3024062

[kfw053-B51] Shipp L. E. Hamdoun A. (2012). ATP-binding cassette (ABC) transporter expression and localization in sea urchin development. Dev. Dyn. 241, 1111–1124. 22473856 10.1002/dvdy.23786 PMC3354041

[kfw053-B52] Smith J. Davidson E. H. (2008). Gene regulatory network subcircuit controlling a dynamic spatial pattern of signaling in the sea urchin embryo. Proc. Natl. Acad. Sci. 105, 20089–20094. 19104065 10.1073/pnas.0806442105 PMC2629318

[kfw053-B53] Todgham A. E. Hofmann G. E. (2009). Transcriptomic response of sea urchin larvae *Strongylocentrotus purpuratus* to CO2-driven seawater acidification. J. Exp. Biol. 212, 2579–2594. 19648403 10.1242/jeb.032540

[kfw053-B54] Vanaverbeke J. Franco M. A. van Oevelen D. Moodley L. Provoost P. Steyaert M. (2008). Benthic responses to sedimentation of phytoplankton on the Belgian Continental Shelf In: Rousseau V Lancelot C Cox D, editors. Current Status of Eutrophication in the Belgian Coastal Zone. Universite Libré de Bruxelles, Bruxelles;. p. 73–90.

[kfw053-B55] Varrella S. Romano G. Costantini S. Ruocco N. Ianora A. Bentley M. G. Costantini M. (2016). Toxic diatom aldehydes affect defence gene networks in sea urchins. PloS ONE 11, e0149734. 26914213 10.1371/journal.pone.0149734 PMC4767821

[kfw053-B56] Varrella S. Romano G. Ianora A. Bentley M. G. Ruocco N. Costantini M. (2014). Molecular response to toxic diatom-derived aldehydes in the sea urchin *Paracentrotus lividus.* Mar. Drugs 12, 2089–2113. 24714125 10.3390/md12042089 PMC4012444

[kfw053-B57] Wetzel R. G. (1995). Death, detritus, and energy flow in aquatic ecosystems. Freshw. Biol. 33, 83–89.

[kfw053-B58] Zito F. Costa C. Scairrino S. Russo R. Angerer L. M. Matranga V. (2003). Expression of univin, a TGF-b growth factor, requires ectoderm-ECM interaction and promotes skeletal growth in the sea urchin embryo. Dev. Biol. 264, 217–227. 14623243 10.1016/j.ydbio.2003.07.015

