# Supplementary material for: First Morphological and Molecular Evidence of the Negative Impact of Diatom-Derived Hydroxyacids on the Sea Urchin Paracentrotus lividus
Source: Toxicol Sci. 2016 Mar 16;151(2):419–33. doi: 10.1093/toxsci/kfw053 (PMC4880139; doi:10.1093/toxsci/kfw053)
Supplement: Supplementary Data [file supp_151_2_419__index.html]

First Morphological and Molecular Evidence of the Negative Impact of Diatom-Derived Hydroxyacids on the Sea Urchin Paracentrotus lividus — First Morphological and Molecular Evidence of the Negative Impact of Diatom-Derived Hydroxyacids on the Sea Urchin Paracentrotus lividus — Supplementary Data 

# First Morphological and Molecular Evidence of the Negative Impact of Diatom-Derived Hydroxyacids on the Sea Urchin *Paracentrotus lividus*

## Supplementary Data

files

- Supplementary Data - zip file
