# Supplementary material for: First Morphological and Molecular Evidence of the Negative Impact of Diatom-Derived Hydroxyacids on the Sea Urchin Paracentrotus lividus
Source: Toxicol Sci. 2016 Mar 16;151(2):419–33. doi: 10.1093/toxsci/kfw053 (PMC4880139; doi:10.1093/toxsci/kfw053)
Supplement: Supplementary Data [file supp_kfw053_suppl_data.zip › toxsci-16-0033-File009.doc]

**Legends to Supplementary Figures**

**Supplementary Figure S1.** Examples of teratogenic *P. lividus* plutei incubated with the four HEPEs (5-, 9-, 11- and 15-HEPE) for 48 hpf (B-J), in comparison with (A) the control (embryos in sea water without HEPEs). Photos were taken with Zeiss Axiovert 135TV, 10x / 0.30 (magnification / numerical aperture). Because the four HEPEs tested induced similar malformations, an overview of the malformations observed has been reported without distinguishing between the HEPEs.

**Supplementary Figure S2.** Examples of (B-K) malformations and (L-M) delayed embryos (still at the pluteus stage but malformed) induced in *P. lividus* embryos observed at one week of incubation with HEPEs at 7.0, 10.0, 15.0 and 30.0 µM in comparison with (A) control embryos, in sea water without HEPEs. Photos were taken with Zeiss Axiovert 135TV, 10x / 0.30 (magnification / numerical aperture).

**Supplementary Figure S3.** Percentage (%) of abnormal ampoules (blue), abnormal plutei (red), normal ampoules (green), normal plutei (yellow) and early plutei (violet) in samples incubated with HEPEs at 7.0, 10.0, 15.0 and 30.0 µM detected at one week after fertilization. Values in graphs represent the mean ± SD (N=3). Significant differences compared to the control (embryos grown in FSW): ***p<0.001. Two-way ANOVA, Bonferroni’s post test (p<0.05).

**Supplementary Figure S4.** Recovery experiments with HEPEs. Percentage (%) of *P. lividus* (A) delayed embryos and (B) abnormal plutei after exposure to different concentrations of HEPEs 7, 10 and 15 μM: 7.0 μM was the concentration used to test the effects of HEPEs on gene expression; treatment with this concentration induced abnormal (about 20%) and delayed (about 15%) plutei; treatments with 10 μM and 15 μM induced a delay in *P. lividus* embryos with very similar percentage of delayed embryos (about 26% and 28%, respectively) but with a very different degree of delay (as reported in Figure 2). Embryos were washed at different times after fertilization, 40 mpf, 2, 5, 9 hpf, corresponding to two-cell stage, 8-cell stage, early blastula and swimming blastula, respectively. The percentages of delayed and abnormal embryos treated with the three different HEPE concentrations and developed up to 48 hpf (no washing) were also reported (grey bar in the histogram). Significant differences compared to the control (no washed embryos): *p<0.05, **p<0.01,***p< 0.001. One-way ANOVA (p< 0.05), with Tukey’s Multiple Comparison Test.

**Supplementary Figure S5.** Addition experiments with HEPEs. Percentage (%) of *P. lividus* (A) delayed embryos and (B) abnormal plutei, produced after adding HEPEs at different concentrations (7.0, 10.0 and 15.0 μM, as in the case of recovery experiments) and times of development: 10 hpf (virgin oocytes), 10 mpf (zygote), 40 mpf (immediately before first cleavage), 2 (8-cell stage), 5 (early blastula) and 9 (hatched blastula) hpf. Significant differences compared to the control (10 mbf): *p<0.05, **p<0.01, ***p<0.001. One-way ANOVA (p<0.05), with Tukey’s Multiple Comparison Test

**Supplementary Figure S6.** The scheme indicate the four functional classes of genes used to which the thirty four genes analyzed in the present study belong: canonical stress genes, genes involved in detoxification processes, genes involved in developmental and differentiation processes and skeletogenic genes.
